# Household food security status and its associated factors among pensioners in Arba Minch town, South Ethiopia

**DOI:** 10.3389/fnut.2024.1363434

**Published:** 2024-04-05

**Authors:** Daniel Niguse Mamo, Kassahun Misgana Worku, Yonas Fissha Adem, Adamu Ambachew Shibabaw, Aklilu Habte, Yosef Haile

**Affiliations:** ^1^Department of Health Informatics, School of Public Health, College of Medicine and Health Science, Arba Minch University, Arba Minch, Ethiopia; ^2^Department of Medical Laboratory Science, College of Medicine and Health Science, Arba Minch University, Arba Minch, Ethiopia; ^3^Department of Public Health, Dessie College of Health Sciences, Dessie, Ethiopia; ^4^Department of Health Informatics, College of Health Science, Mettu University, Mettu, Ethiopia; ^5^School of Public Health, College of Medicine and Health Science, Wachemo University, Hossana, Ethiopia; ^6^Department of Public Health, School of Public Health, College of Medicine and Health Science, Arba Minch University, Arba Minch, Ethiopia

**Keywords:** food insecurity, pensioners, Arba Minch town, Ethiopia, Gamo zone

## Abstract

**Introduction:**

Food insecurity has remained a serious public health problem in developing countries, such as Ethiopia, over the past two decades. Vulnerable populations, such as pensioners, have been affected by this problem because of emerging socio-demographic changes, a global financial crisis, and climate change, all of which have contributed to the high food prices. Hence, this study aimed to assess household food security status and associated factors among pensioners in Arba Minch town, South Ethiopia.

**Methods:**

A community-based cross-sectional study design was conducted from September to October 2023. Two hundred forty-four pensioners were chosen using a simple random sampling technique. Data were collected, cleaned, and entered into EPI-Data version 4.6 and exported to SPSS version 25 for analysis. Variables with a *p*-value of ≤0.25 in the bivariate analyses were candidates for the multivariable regression analysis. In the multivariable logistic regression, variables with a *p*-value of 0.05 were considered to have a significant association with the dependent variable.

**Results:**

A total of 238 retired people were interviewed, with a response rate of 97.5%. Among the interviewed pensioners, 223 (91.4%) households were food insecure. Having more than one dependent member [AOR = 2.4, 95% C.I: 1.30, 6.64], being jobless after retirement [AOR = 3, 95% C.I:1.17, 5.61], and being in the lowest tertile of wealth status [AOR = 2, 95% C.I:1.36, 4.99] were identified as predictors of food insecurity.

**Conclusion:**

The magnitude of household food insecurity was higher compared to the national average, and factors such as the current occupational status of the household head, dependency ratio, and wealth status of the household were significantly associated with household food insecurity. Therefore, policymakers and programmers should provide new strategies focusing on additional income-generating activities and salary increments and consider free services such as school fees and healthcare.

## Introduction

Food security occurs when everyone has constant access to sufficient, safe, wholesome, and nutritious food to suit their dietary needs and food preferences for an active and healthy lifestyle (1). One of the main problems in recent decades has been the issue of global food security. In recent years, plans to achieve food security in developing countries have needed to be completely revised due to the rising cost and scarcity of resources, such as food, land, and water, (2).

In 2017, undernourishment, or persistent food insecurity, plagued almost 821 million people worldwide. The regions most impacted by this public health issue were countries in South America and the majority of countries in Africa. To ensure that we “leave no one behind” on the path to a world with zero hunger, there is a lot of work to be done, and growing hunger and food insecurity are warning signs (3). In sub-Saharan Africa, an estimated 23.2% of the population, or one in four to one in five people, may have experienced chronic food insecurity in 2017 (4).

Even though it is anticipated that by 2050 there will be more than 50 million people living in cities, assessments of Ethiopia’s food security and vulnerability have largely focused on rural areas (5). Studies indicate that in Ethiopia, between 80 and 84% of pensioners lack access to food (6, 7). Despite the rapid increase in the world’s elderly population, few low- and middle-income countries have focused on the dietary difficulties of pensioners (8). As a result, low-income people, especially those in developing countries, are vulnerable to economic volatility and change despite recent efforts made globally to reform pensions (9).

Through initiatives such as the Productive Safety Net Program and pension plans, the government of Ethiopia is assuming a strong leadership position in the fight against food insecurity (10). However, it is difficult to comprehend the selection criteria and the procedure by which people are prioritized to enroll in the Productive Safety Net Program. The monthly income pensioners receive is allocated for different purposes, such as food, health, utilities, other households, saving or investing, education, and social participation. However, this allocation may lead to food insecurity among pension users. Additionally, the major concern of retirees is the rate of galloping inflation that of price of goods in the market (9).

Research on food security in developing nations focuses mostly on rural areas, and there is a propensity to see food insecurity as a problem that primarily affects rural areas in these nations (11–14). Many studies look at the severe household food insecurity in rural Ethiopia and its contributing variables, and most of them concentrate on general families rather than vulnerable groups such as pensioners (15–18). The current situation in the country, including civil war, drought, and the inflation rate, can affect this vulnerable group. Given the facts mentioned above, this study focused on assessing food insecurity and associated factors among pensioners in Arba Minch town, South Ethiopia.

## Methods and materials

### Study design and period

A community-based cross-sectional study was conducted on household food insecurity and its associated factors among pensioners in Arba Minch town, South Ethiopia, from September to October 2023.

### Study area

The study was conducted in Arba Minch town, which is the administrative center of the Gamo zone found in the southern part of Ethiopia. The town is located at a distance of 505 km from Addis Ababa and 275 km from Hawassa. The city was founded in 1962, when the capital of Gamo Gofa province was transferred from Chencha to Arba Minch town. Since then, it has served as the capital of Gamogofa province and is currently the capital of Gamo zone.

### Study population

All pensioners who resided in Arba Minch town were the study population.

### Inclusion and exclusion criteria

All pensioner households that had been in Arba Minch town, South Ethiopia, for at least 6 months before the recruitment period were chosen for inclusion, while any individual who became seriously ill during the study period was excluded.

### Sample size determination

The sample size was determined by using a single population proportion formula considering the 82.5% prevalence of household food insecurity among pensioners from a study conducted in Debre Markos (6), a 95% confidence interval (CI), and a 5% margin of error (d).

n=Zα22P1−Pd2

$$n=\frac{{\left(1.96\right)}^{2}*0.825\left(1-0.825\right)}{{\left(0.05\right)}^{2}}=222$$

where *n* = minimum sample size.

*Z* = 1.96, normal deviant at the portion of the 95% confidence interval of a two-tailed test.

*p* = 82.5%, prevalence of household food insecurity from a study conducted in Debre Markos, Ethiopia.

*d* = margin of error acceptable were taken as 5% = 0.05.

The minimum required sample size, as calculated, was 222.

By adding 10% for non-response, the final sample size was 244.

### Sampling procedure

The pensioners’ registration data were obtained from the Labor and Social Affairs office. By using the registration as a sampling frame, we selected the households by using simple random sampling (lottery method). Finally, we contacted each household with the help of local guides, using a list of study participants’ names and phone numbers collected from registration. We also referenced their specific kebele and house numbers as documented in retirees’ registration records.

### Data collection tool and technique

Data were collected by an interviewer-administered semi-structured questionnaire. The tool had three parts: socio-demographic characteristics of the respondents, economic resources and assets of the household, and measures of household food insecurity access score. The questionnaire on household food security status was adopted from the Food and Nutrition Technical Assistance (FANTA) indicator guide version 3, the Household Food Insecurity Access Scale (19).

Three enumerators who are familiar with the study sites were trained and recruited to conduct home-to-home visits. Local assistants, one for each site, were recruited to facilitate the identification of households and arrange appointments for interviews. Information related to the background characteristics and food consumption of the household was collected from an adult member of the household available at home during the visit. Household wealth status was assessed using wealth constructs reflecting household assets and utilities adopted from the Ethiopian Demographic and Health Survey.

### Study variable

The dependent variable is food insecurity, and the independent variables are socio-demographic and economic factors such as educational status, sex, age, family size, ownership of a bank account, support from relatives, source of household income, asset ownership, access to credit institutions, and health-related factors (self-reported household head health status).

### Operational definition

#### Food insecure households

According to the Household Food Insecurity and Access Scale (HFIAS), households with scores of 0–1 were categorized as food secure, while those with scores of 2 and above were considered food insecure. This categorization was clearly described in the data analysis section (19).

#### Monthly household income

Monthly household income refers to what members of a household earn from permanent employment or daily labor; economic activities such as farming and small businesses; and rental income.

#### Asset ownership

Asset ownership refers to the household’s possession of durable assets that are the most relevant for food security outcomes, such as household appliances, valuables, farming land in rural areas, and livestock.

#### Source of household income

Source of household income refers to the household’s income source, which could be from pensions or other sources, such as renting or small businesses.

#### Dependency ratio (dependent member)

Dependent member refers to children less than 15 years old in the households of pensioners.

#### Employee

In this study, “employees” refers to retired people who were formerly employed in government and private organizations. However, these organizations are different from the earlier organizations in which they had worked in before. In addition, those pensioners who were working as security guards were not considered employees in our study.

### Data quality assurance

To ensure the quality of the data before data collection, a semi-structured questionnaire was prepared in English and translated into the local language (Amharic). To ensure the quality of the data and to make sure that all assessment team members can administer the questionnaires properly, training for data collectors and supervisors was given.

An internationally standardized tool known as HFIAS was used in this study. A pre-test was carried out on 5% of the study participants in Birbir Town pension user households to identify issues and take corrective actions to the contents of the questionnaire. The data collectors and supervisors were university graduate BSc and MSc holders, respectively. At the end of every data collection day, each questionnaire was examined for completeness and consistency by the supervisors and the principal investigator, and pertinent feedback was given to the data collectors.

### Data processing and analysis

Data were entered using Epidata version 4.6 after proper coding and checking and then exported to SPSS version 25 for analysis. Data were explored using exploratory analysis to check the levels of missing values and the presence of influential outliers, and pseudo-regression was carried out to check for multi-co-linearity. Descriptive statistics were used to describe the data. The wealth index was constructed using principal component analysis (PCA) from 27 items. After all assumptions of PCA were checked, the household wealth status was ranked into three tertiles.

Cross-tabulation and bivariate logistic regression analyses were conducted to examine the association between individual explanatory and outcome variables. Variables with a *p*-value of <0.25 were candidates for multivariable logistic regression (20). An odds ratio with 95% confidence intervals was used to measure the strength of the association between dependent and independent variables. Model fitness was good according to Hosmer and Lemshow’s goodness of fit test (*p* = 0.58). A *p*-value of <0.05 was used to declare the level of statistical significance.

According to FAANTA III –UNICEF, HFIAS is a tool used to estimate the food insecurity of households in the United States each year. It has nine questions within it. The Household Food Insecurity Access category for each household is classified as 1 = food secure, 2 = mildly food insecure access, 3 = moderately food insecure access, and 4 = severely food insecure access. The Household Food Insecurity and Access (HFIA) category = 1 if [(Question 1a = 0 or Q1a = 1) and Q2 = 0 and Q3 = 0 and Q4 = 0 and Q5 = 0 and Q6 = 0 and Q7 = 0 and Q8 = 0 and Q9 = 0]. The HFIA category = 2 if [(Question 1a = 2 or Q1a = 3 or Q2a = 1 or Q2a = 2 or Q2a = 3 or Q3a = 1 or Q4a = 1) and Q5 = 0 and Q6 = 0 and Q7 = 0 and Q8 = 0 and Q9 = 0]. The HFIA category = 3 if [(Q3a = 2 or Q3a = 3 or Q4a = 2 or Q4a = 3 or Q5a = 1 or Q5a = 2 or Q6a = 1 or Q6a = 2) and Q7 = 0 and Q8 = 0 and Q9 = 0]. The HFIA category = 4 if [Q5a = 3 or Q6a = 3 or Q7a = 1 or Q7a = 2 or Q7a = 3 or Q8a = 1 or Q8a = 2 or Q8a = 3 or Q9a = 1 or Q9a = 2 or Q9a = 3].

Responses to the nine HFIAS questions were summed using SPSS version 25 to create a household food security score, with a minimum of “0” and a maximum score of “27.” Finally, those households with HFIA categories 2, 3, and 4, or mild food insecure, moderate food insecure, and severe food insecure, were categorized as food insecure households, and HFIA category 1 was categorized as food secure households for further analysis using logistic regression.

## Results

### Socio-demographic characteristics

Two hundred thirty-eight pensioners were interviewed, yielding a response rate of 97.5%. Approximately 42.2% of respondents were spouses, while 40.2% were the heads of the household. More than three-fourths of households (82.4%) were headed by men. Approximately 109 (44.7%) household heads were > 65 years of age. The average (±SD) age of the household head was 62 (±9.3) years. The average household size of all the respondents was approximately 4, which ranged from a minimum of 2 to a maximum of 6 people per household. In terms of educational status, 77.9% of the respondents had a certificate, and more than 22.68% of households were jobless after retirement (Table 1).

**Table 1:** Socio-demographic characteristics of pensioner households in Arba Minch town, South Ethiopia, 2023 (*n* = 238).

Characteristics	Frequency (*n* = 238)	Percentage (%)
Age of HH head		
40–44	11	4.6
45–49	17	7.14
50–54	11	4.6
55–59	18	7.56
60–64	73	30.67
>65	108	45.37
Sex of HH head		
Male	196	82.3
Female	42	17.7
Ethnicity		
Oromo	188	78.99
Amhara	31	13.02
Other	19	7.99
Marital Status		
Married	222	93.27
Divorced	4	1.68
Widowed	12	5.05
Educational level of HH head		
Unable to read and write	1	0.42
Able to read and write	2	0.84
Primary school	14	5.88
Secondary school	35	14.7
Certificate and above	186	78.15
Respondent relationships with HH		
Head	97	40.75
Spouse	101	42.43
Son/daughter	28	11.76
Relative	12	5.04
Number of permanent HH members		
1–3	58	24.4
4–6	180	75.6
Number of dependent members		
0	78	32.7
= >1	160	67.3
Current occupational status		
Employed	5	2.1
Merchant	97	40.75
Daily labor	29	12.2
Guard	50	21
Jobless	54	22.68

### Economic resource of household

The major source of income, in addition to pension, was support from a son/daughter or relative for 83 (34%) households, while renting and small businesses were major sources of income for 66 (27%) and 27 (11.1%) households, respectively. More than half of households—133 (55.8%)—had no access to micro and small business enterprises (MSBEs) in the last year. All pensioner households had savings (bank accounts). The majority of households, 168 (70.5%), were living in their own houses, while 54 (22.68%) pensioners rented from the government, and 16 (6.7%) were living in houses rented from private sources. Based on the wealth index analysis, 62 (26%) of pensioner households were in the lowest tertile, 98 (41.2%) were middle, and 78 (32.7%) were in the highest tertile (Table 2).

**Table 2:** Economic resource of pensioners in Arba Minch town, South Ethiopia, 2023 (*n* = 238).

Variables	Frequency *N* = 238	Percentage (%)
Major source of income		
Pension only	68	28.57
Small business	24	10.08
Renting income	66	27.73
Support from son/daughter or relatives	80	33.61
Access to micro and small business enterprise (MSBE)		
Yes	105	44.1
No	133	55.8
Owner of bank account		
Yes	238	100
No	0	0
Owner of the house		
Owned by the household	194	81.5
Rented from government	38	15.96
Rented from private	6	2.52
Wealth index		
Lowest tertile	62	26
Middle tertile	98	41.2
Upper tertile	78	32.8

### Household food security status

The household data were used to provide a descriptive analysis of the food security situation based on the Household Food Insecurity Access Scale tool.

**Figure 1:**
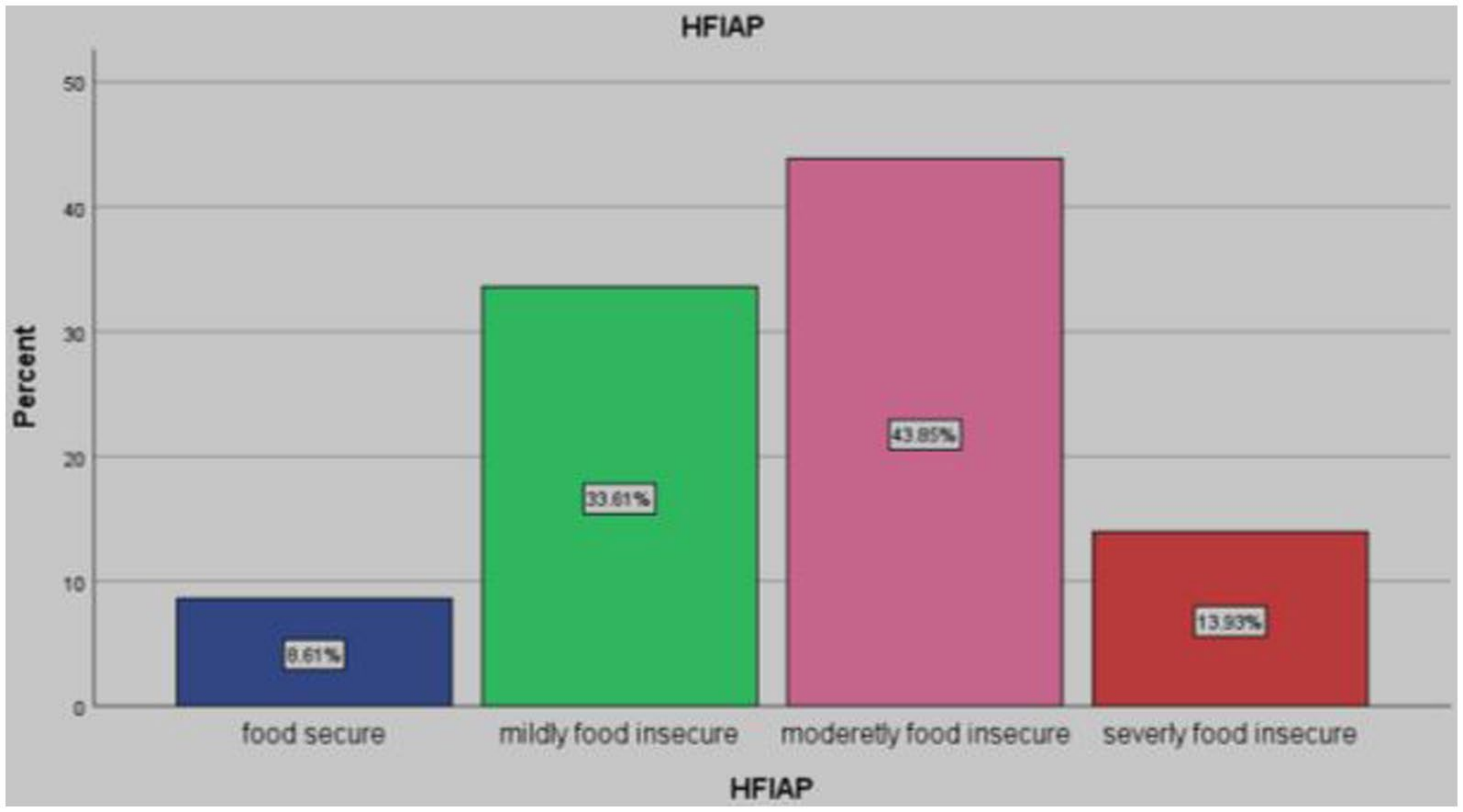
Household food security status category of pensioners in Arba Minch town, Southern Ethiopia, 2023.

According to HFIAS, 21 (8.6%) households were food secure, and 223 (91.4%) were food insecure. Of the food insecure households, 82 (33.6%) were classified as mildly food insecure 107 (43.9%) households were classified as moderately food insecure, and 34 (13.9%) households were classified as severely food insecure (Figure 1).

### Factors associated with household food security status

In binary logistic regression analysis, gender, current occupational status, size of household, dependency ratio, access to MSBE, household income, health status, and house owner were selected as candidate variables for the multivariable logistic regression. The multivariable logistic regression analysis revealed that the current occupational status of a household, the number of dependent members (dependency ratio), and wealth status were significantly associated with household food security status.

The results of a multivariable analysis revealed that the odds of households with a currently jobless head were three times [AOR = 3; 95 CI (1.17, 5.61)] more likely to be food insecure than those currently employed in the private and non-profit sectors. The odds of households with a lower wealth index were two times [AOR = 2; 95 CI: (1.36, 4.99)] more likely food insecure when compared to households with a higher wealth index. Dependency ratio is also an independent predictor of food security status, the odds of households with dependent members equal to one or greater than one were two times [AOR = 2.4; 95 CI: (1.30, 6.64)] more likely to be food insecure when compared to a household with no children (Table 3).

**Table 3:** Factors associated with household food security status among pensioners in Arba Minch town, South Ethiopia, 2023 (*n* = 238).

Predictors	Food insecure		COR [95% CI]	AOR [95% CI]	*p*-value
	Yes	No			
Sex					
Male	176	20	1	1	
Female	39	3	4.43 [0.02, 0.54]*	1.875 [0.86, 1.03]	0.66
Current occupational status					
Employed	3	2	1	1	
Small business	88	9	6.5 [1.19, 9.37]*	6.62 [0.92, 1.15]	0.582
Daily labor	28	1	18.6 [0.93, 4.734]	14.23 [0.93, 1.20]	0.89
Guard	48	2	16 [1.67, 4.98]*	16.2 [0.46, 11.23]	0.99
Jobless	50	4	8.3 [2.34–19.67]	3[1.17, 5.61]	0.019**
Dependency ratio					
0	64	14	1	1	
≥1	153	7	4.7 [2.14, 7.94]	2.4 [1.30, 6.64]	0.03**
Access to micro and small business enterprise (MSBE)					
Yes	87	18	1	1	
No	130	3	0.111 [0.12, 0.24]*	0.129 [0.94, 1.318]	0.134
Wealth index					
Lowest tertile	58	4	2.13 [1.18, 3.43]	2 [1.36, 4.99]	0.032**
Middle tertile	91	7	1.91 [3.60, 10.46]	1.9 [0.76, 1.02]	0.341
Highest tertile	68	10	1	1	
Household Size					
1–3	50	8	1	1	
4–6	167	13	2 [0.03, 0.037]*	2.01 [0.984, 1.105]	0.423
Health status of HH head					
Good	98	13	1	1	
Fair	95	7	1.8 [0.15, 0.276]*	1.257 [0.95, 1.071]	0.257
Bad	24	1	3.18 [0.02, 0.2]*	3 [0.736, 1.048]	0.383
Owner of the house					
Owned by HH	150	18	1		
Rented from government	52	2	3.12 [2.17, 6.94]*	2.78 [0.73, 1.042]	0.421
Rented from private	15	1	1.8 [3.90, 13.12]*	1.8 [0.96, 1.11]	0.372

*significant at a *p*-value of ≤ 0.25.

**significant at a *p*-value of ≤ 0.05.

## Discussion

According to the household food insecurity access prevalence (HFIAP) score, 91.4% [95% CI: 86.4, 94.6] of pensioner households were food insecure. The findings are considerably higher than the level of national food insecurity (20.5%) reported in the World Food Program report of 2016. In line with this study, high levels of food insecurity have also been documented in households conducted in areas adjacent to the wetlands in Uganda (93%) (21). The findings were higher than the proportion of food insecurity among pension users in Jimma and Debre Markos towns, which were 83.5 and 82.5%, respectively. The level of household food insecurity reported in the current study is also higher than in a previous study conducted in the Kanchanpur district of far-western Nepal (41.1%) (22), and in Tehran, Iran (56.9%) (23).

The significant rate of food price inflation in the nation over the previous 12 months may help to explain the study’s high level of food insecurity. Additionally, households’ heavy reliance on food purchased from the market, fluctuating food costs, the spread of COVID-19, and political unrest and war in various parts of the nation all contributed to the greater food insecurity experienced by study participants. In this study, homes with one or more dependent members (children under the age of 15) had a higher likelihood of experiencing food insecurity than households without children under the age of 15. This result concurred with studies from Tehran, Iran, and the Kanchanpur area in far-western Nepal (22, 24). This is mostly because children under the age of 15 are not productive, which would result in increased food insecurity as more people depend on an inadequate income to survive.

Furthermore, our study illustrated a significant association between household food insecurity and the wealth status of pension beneficiaries, such that pension beneficiaries with a lower wealth index were more likely to be food insecure as compared to pension users with a higher wealth index. This finding was supported by studies conducted in Addis Ababa (25). This might be because the high asset value of the household increases the purchasing power of household consumption items since pension users primarily purchase food from the market. Furthermore, income determines how much can be spent on various needs of the household (26).

In addition, the occupational status of the household head was another predictor of food insecurity among the study population. Households headed by those who are currently jobless were more likely to be food insecure than households that are currently employed in private and NGO sectors. This result was consistent with a previous study in Jimma Town (27) and a recent investigation of home food insecurity and related variables in Wolayita Sodo, Southern Ethiopia, which discovered a strong relationship between the household’s work position and household food insecurity (28). Additionally, research in Nigeria lends credence to it. Self-employment prospects may assist households to diversify and grow their income, and heads of households who are actively employed make more money and have a better ability to buy food than unemployed people or retirees who entirely rely on their pensions (29).

### Limitations of the study

The potential limitation of this study was a recall and social desirability bias, though an attempt was made to minimize it by clarifying the purpose of the study. The other limitation was that we could not establish causal relationships between the independent and dependent variables because of the cross-sectional nature of the study design. The self-report was used to determine individual health status, which may not exactly indicate their health status.

## Conclusion

In this study, the majority of pensioner households were food insecure. The findings of this study showed that household food insecurity among pensioners was associated with socio-economic factors such as wealth index, dependency ratio, and the occupational status of the head of the household. The Town Health Bureau should take action based on increased awareness of the effects of population growth at the family, community, and national levels. This could lead to lower fertility and more spaced births, resulting in a decreased dependency ratio and household size. Policymakers and programmers should provide new strategies focusing on the Urban Safety Net Program for lower-income households, additional income-generating activities, salary increments, and free services such as school fees and healthcare.

## Data availability statement

The raw data supporting the conclusions of this article will be made available by the authors, without undue reservation.

## Ethics statement

The studies involving humans were approved by Ethical review committee of Arba Minch University, College of Health and medical science. The studies were conducted in accordance with the local legislation and institutional requirements. The participants provided their written informed consent to participate in this study.

## Author contributions

DM: Formal analysis, Investigation, Writing – original draft. KW: Data curation, Formal analysis, Writing – review & editing. YA: Data curation, Software, Writing – original draft. AS: Data curation, Formal analysis, Visualization, Writing – review & editing. AH: Data curation, Formal analysis, Validation, Writing – review & editing. YH: Formal analysis, Methodology, Writing – review & editing.
